# Digital Cognitive Behavioral- and Mindfulness-Based Stress-Management Interventions for Survivors of Breast Cancer: Development Study

**DOI:** 10.2196/48719

**Published:** 2023-09-19

**Authors:** Elin Børøsund, Anders Meland, Hege R Eriksen, Christine M Rygg, Giske Ursin, Lise Solberg Nes

**Affiliations:** 1 Department of Digital Health Research Division of Medicine Oslo University Hospital Oslo Norway; 2 Department of Nursing and Health Sciences Faculty of Health and Social Sciences University of South-Eastern Norway Drammen Norway; 3 Department of Sport and Social Sciences School of Sport Sciences Oslo Norway; 4 Department of Sport Food and Natural Sciences Western Norway University of Applied Sciences Bergen Norway; 5 Cancer Registry of Norway Oslo Norway; 6 Department of Preventive Medicine Keck School of Medicine University of Southern California Los Angeles, CA United States; 7 Department of Nutrition Institute of Basic Medical Sciences University of Oslo Oslo Norway; 8 Institute of Clinical Medicine Faculty of Medicine University of Oslo Oslo Norway; 9 Department of Psychiatry and Psychology College of Medicine and Science Mayo Clinic Rochester, MN United States

**Keywords:** cancer, stress management, mindfulness, cognitive behavioral therapy, digital, eHealth, mHealth, app, user-driven development, usability

## Abstract

**Background:**

Psychosocial stress-management interventions can reduce stress and distress and improve the quality of life for survivors of cancer. As these in-person interventions are not always offered or accessible, evidence-informed digital stress-management interventions may have the potential to improve outreach of psychosocial support for survivors of cancer. Few such digital interventions exist so far, few if any have been developed specifically for survivors of breast cancer, and few if any have attempted to explore more than 1 distinct type of intervention framework.

**Objective:**

This study aimed to develop 2 digital psychosocial stress-management interventions for survivors of breast cancer; 1 cognitive behavioral therapy-based intervention (CBI), and 1 mindfulness-based intervention (MBI).

**Methods:**

The development of the CBI and MBI interventions originated from the existing StressProffen program, a digital stress-management intervention program for survivors of cancer, based on a primarily cognitive behavioral therapeutic concept. Development processes entailed a multidisciplinary design approach and were iteratively conducted in close collaboration between key stakeholders, including experts within psychosocial oncology, cancer epidemiology, stress-management, and eHealth as well as survivors of breast cancer and health care providers. Core psychosocial oncology stress-management and cancer epidemiology experts first conducted a series of workshops to identify cognitive behavioral and mindfulness specific StressProffen content, overlapping psychoeducational content, and areas where development and incorporation of new material were needed. Following the program content adaptation and development phase, phases related to user testing of new content and technical, privacy, security, and ethical aspects and adjustments ensued. Intervention content for the distinct CBI and MBI interventions was refined in iterative user-centered design processes and adjusted to electronic format through stakeholder-centered iterations.

**Results:**

For the CBI version, the mindfulness-based content of the original StressProffen was removed, and for the MBI version, cognitive behavioral content was removed. Varying degrees of new content were created for both versions, using a similar layout as for the original StressProffen program. New content and new exercises in particular were tested by survivors of breast cancer and a project-related editorial team, resulting in subsequent user centered adjustments, including ensuring auditory versions and adequate explanations before less intuitive sections. Other improvements included implementing a standard closing sentence to round off every exercise, and allowing participants to choose the length of some of the mindfulness exercises. A legal disclaimer and a description of data collection, user rights and study contact information were included to meet ethical, privacy, and security requirements.

**Conclusions:**

This study shows how theory specific (ie, CBI and MBI) digital stress-management interventions for survivors of breast cancer can be developed through extensive collaborations between key stakeholders, including scientists, health care providers, and survivors of breast cancer. Offering a variety of evidence-informed stress-management approaches may potentially increase interest for outreach and impact of psychosocial interventions for survivors of cancer.

**International Registered Report Identifier (IRRID):**

RR2-10.2196/47195

## Introduction

### Background

Survivors of cancer encounter a multitude of physical and psychosocial challenges and demands, naturally impacting well-being and quality of life (QoL) [1-3]. Coping can be difficult [2,4], and psychosocial stress-management interventions, such as cognitive behavioral therapy (CBT) [1,5-9] and more recently also mindfulness-based interventions (MBIs) [10-13] for survivors of cancer have shown to be of benefit, for example, in terms of reducing distress, stress, anxiety, and depression as well as improving QoL [1,5-13]. Such in-person interventions are however not always available, and many access barriers exist, including geographical distance, issues with transportation or finances, and survivors of cancer may not always feel well enough to attend in-person interventions [14]. Digital solutions may therefore be 1 way to improve access and outreach of evidence-informed psychosocial support for survivors of cancer.

eHealth interventions for survivors of cancer exist, but findings are still somewhat mixed and inconclusive [15-21]. Theoretically based, end user (ie, survivors of cancer) and other key stakeholders (eg, health care providers) involved development, with feasibility as well as efficacy testing through randomized controlled trials, are therefore needed [15-19].

A recently developed user-centered, feasibility- and efficacy tested digital stress-management intervention for survivors of cancer, *StressProffen* [22], has been described by survivors of cancer as an appreciated and easily accessible stress-management tool [23]. A randomized controlled trial (RCT) showed survivors of cancer (ie, 48% survivors of breast cancer) receiving *StressProffen* over 12 months, compared to usual care controls, to experience reduced anxiety, depression and self-regulatory fatigue, and improved health related QoL [24,25]. *StressProffen* is considered a cognitive-behavioral stress-management intervention [22-25], but does, as with other established cognitive behavioral stress-management interventions [26], also include aspects of additional theoretical psychosocial approaches, including mindfulness.

While both CBT and mindfulness have indications of being effective psychosocial interventions for survivors of cancer, reviews examining interventions to improve psychological well-being and QoL in patients with cancer have pointed to CBT as the gold standard intervention with evidence of effect, and the evidence of MBIs being more mixed [27], with existing mindfulness-based studies potentially experiencing methodological limitations [27,28]. For example, a systematic review of systematic reviews examining a number of rehabilitation interventions following breast cancer, found convincing material (ie, several meta-analyses and systematic reviews) of CBT interventions having positive impact on anxiety, depression, QoL, mood disturbance, body image, sleep disturbances, and self-esteem. However, only 1 meta-analysis indicated that mindfulness-based stress reduction could also improve anxiety, depression, stress, and overall QoL [29]. Other reviews, for example, examining impact of CBT and mindfulness on sleep disturbances in survivors of cancer, have found both CBT and mindfulness for insomnia to be effective [30], although with CBT often being pointed to as having the strongest effect and the findings for MBIs being somewhat more inconsistent [28]. There are however studies pointing to MBIs as effective in coping with chronic health disorders including cancer [11,12,31], and a recent systematic review found mindfulness-based stress reduction interventions to be less costly and more effective when compared with the usual care of CBT among patients with health challenges including breast cancer [32].

To date, digital self-management interventions examining and comparing asynchronous cognitive behavioral and mindfulness intervention approaches for survivors of cancer are at best scarce, even though the preference and suitability of these interventions may vary for the potential end users.

### Objectives

This study aimed to develop 2 distinct asynchronous digital psychosocial stress-management intervention programs for survivors of breast cancer, based on the existing *StressProffen* intervention; one cognitive behavioral therapy-based intervention (CBI) and one MBI.

## Methods

### Study Design

The development process contained a multidisciplinary design approach with elements from user-centered design.

### The Original StressProffen Intervention Program

*StressProffen* is a digital evidence-based stress-management intervention program for survivors of cancer, delivered as an app, developed by the Department of Digital Health Research at Oslo University Hospital. The original *StressProffen* program was designed, developed, and tested (ie, feasibility and efficacy) in close collaboration between researchers, eHealth experts, privacy and security experts, psychosocial-oncology health care providers, and survivors of cancer [22-25]. The program contains 10 primarily cognitive-behavioral stress-management based modules, with aspects from mindfulness. For details on design, development, and usability testing, please see Børøsund et al [22] in 2018.

The original *StressProffen* program modules cover a variety of themes, including (1) *what is stress*; (2) *stress, QoL, and planning*; (3) *thoughts, feelings, and self-care*; (4) *mindfulness, rational thought replacement, and guided imagery*; (5) *stress and coping*; (6) *social support, humor, and meditation*; (7) *anger management and conflict style awareness*; (8) *assertiveness and communication*; (9) *health behaviors and setting goals*; and (10) *review and summary.* Each module consist of separate steps (ie, 9 to 14 steps), and contains a combination of psychoeducational material, suggestions, words of wisdom and exercises (eg, diaphragmatic breathing, mindfulness, progressive muscle relaxation, and thought challenges). Users of the program can at any point choose between reading or listening (ie, written content spoken verbatim). To encourage reflection and practice there is a 3-day delay after the user has completed 1 module before being able to access the next module. To provide structure and allow for individualization, the 4 first modules are sequential, while the order of modules 5-9 can be individually chosen. Module 10 provides a review and suggestions for “the road ahead.”

### Adaptation and Development of 2 Distinct Digital Interventions—StressProffen CBI and MBI

#### Overview

Aiming to use the existing *StressProffen* as a basis to develop 2 interventions, the goal was for the content of the interventions to be the main differing characteristic, developing and delivering the CBI and MBI versions in an identical format with relatively similar complexity. The core content development group (LSN, AM, HRE, and GU) met in biweekly to monthly workshops to examine and decide on specific content to include or exclude in the development of the new *StressProffen* versions. An expanded content development team (GU, LSN, AM, HRE, EB, and CMR) then continued to meet in biweekly to monthly workshops during the next phase to discuss content and adjustments needed for the new CBI- and MBI-versions. In addition, the larger project group, consisting of the expanded project content group, breast cancer experts, national and international psychosocial oncology-, CBT- and mindfulness-experts, a user representative (ie, representing women with breast cancer) and eHealth experts met twice and discussed aspects of the new interventions. The development process consisted of the steps described below.

#### Program Content—Adaptation and Development

To ease the comparison of the content of each section, the *StressProffen* original content was first transferred to a Word (Microsoft Corp) document with the pages split in 2 separate columns. Identical text was included for each section in each column to allow for easy comparison of content. Further, 4 experts within stress-management, CBT, mindfulness, and cancer epidemiology (LSN, AM, HRE, and GU), the core content development group, first independently reviewed the text and placed the content in the CBI or MBI columns based on their personal evaluation of the content. All members of the group used the “track changes” function in Microsoft Word, and then met to discuss steps for further project progress. General psychoeducational content was discussed and primarily kept in both versions (ie, CBI and MBI). For the distinct CBI-version, mindfulness-based content was identified and discussed for potential deletion or adjustment. For the MBI-version, CBT-based content was identified and discussed for potential deletion or adjustment. Need for additional modifications or new material for either of the 2 distinct versions was also identified and a detailed plan for further development formed.

To ensure harmonized interventions related to module length, number of intervention or module steps and number of exercises, new material was developed (eg, when deleting mindfulness-based content from CBI or vice versa) by the 4 core experts (LSN, AM, HRE, and GU). The adjustment of existing and development of new CBI-based content was led by LSN and HRE, while the adjustment of existing and development of new mindfulness-based content was led by AM. To ensure easily understandable language and fit with the original *StressProffen* program, any new content was reviewed by members of the content editorial team at the Department of Digital Health Research, and uncertainties discussed within the expanded content development team (LSN, AM, HRE, GU, EB, and CMR) and with user representatives when deemed necessary. To calculate proportion of content retained, adjusted, or removed, module steps in the new versions were compared with module steps in the original *StressProffen* program.

#### User Testing of New Exercises

As the original *StressProffen* content has already been user tested by survivors of cancer as well as health care providers [22], only new, developed content was user tested in the 2 new versions. To ensure user-friendliness of particularly new exercises, women with breast cancer were invited to test any new exercises developed for the 2 distinct versions. The project user representative invited women in breast cancer groups (eg, local organizations) to participate in user testing, and women interested in participating were given oral and written information and provided written informed consent prior to participation. In addition, a project-related editorial team at the Department of Digital Health Research conducted testing of new content and particularly exercises.

#### Technical and Static Aspects and Adjustments

The original *StressProffen* program also contains static pages with reminders, “quotes of the day,” and privacy and security information. To ensure that these static pages were in line with what is to be expected in the more “pure” CBI and MBI interventions, the core content development group (LSN, AM, HRE, and GU) reviewed the static text independently, applied the “track changes” function in Microsoft Word, met to discuss findings, and then reached decisions regarding removal, modification, or addition of content for the 2 versions.

### Ethics Approval

The most recent version of the *StressProffen* technical platform, already approved by the hospital privacy and security protection committee, was used for the development of the *StressProffen* CBI- and MBI-versions, and the 2 new program versions were subsequently approved through the existing organizational *StressProffen* risk assessment. All user testing procedures were approved by the hospital privacy and security protection committee (18/13312). The participating women with breast cancer provided written informed consent prior to user testing, and received a gift card equivalent of US $30 as compensation for their time and for travel expenses involved. Data from user testing (written notes) were stored deidentified at a secure server at the hospital.

## Results

### Program Content—Adaptation and Development

**Figure 1:**
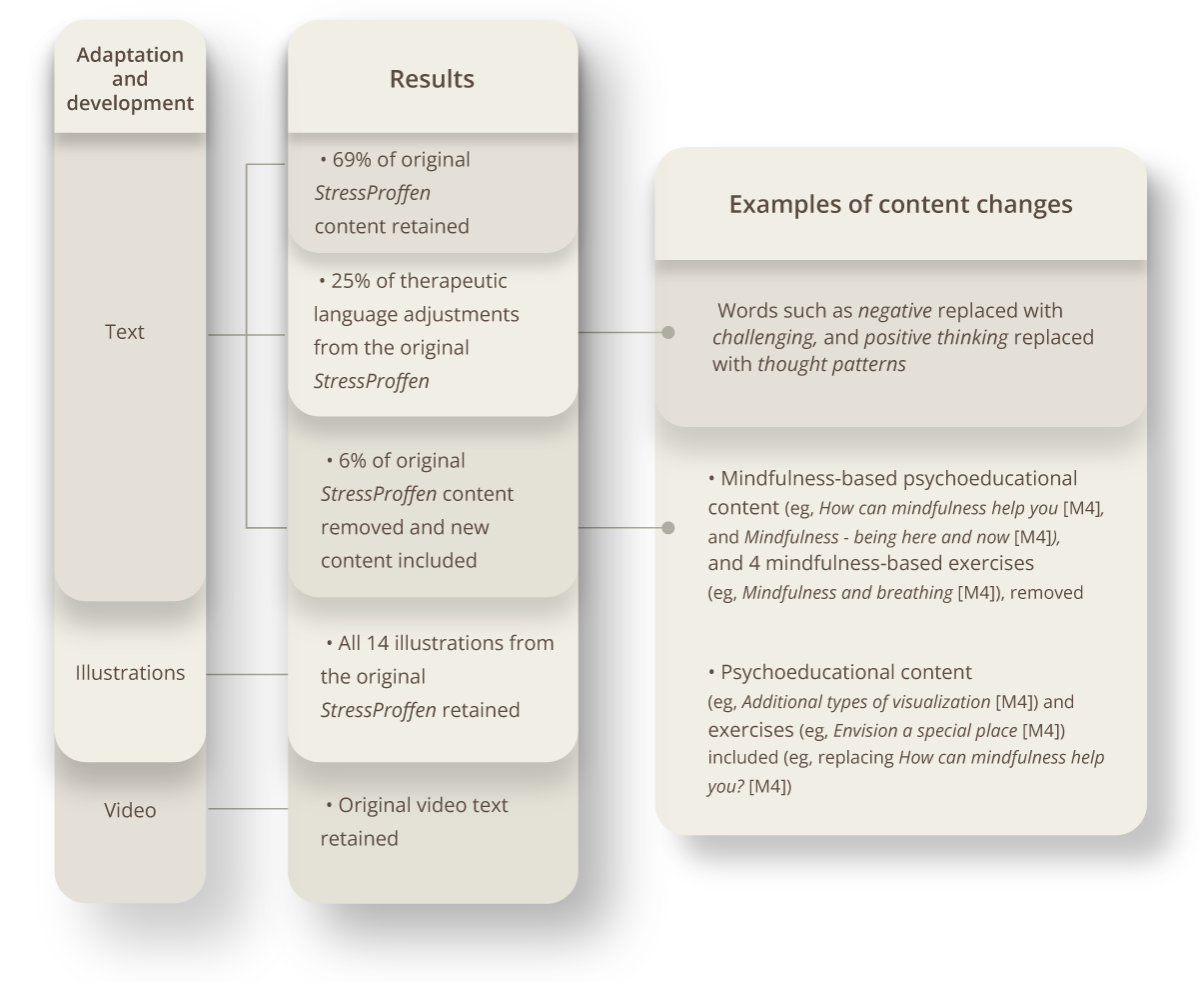
Overview—StressProffen CBI. Program content adaptation and development. CBI: cognitive behavioral therapy-based intervention; M: module.

**Figure 2:**
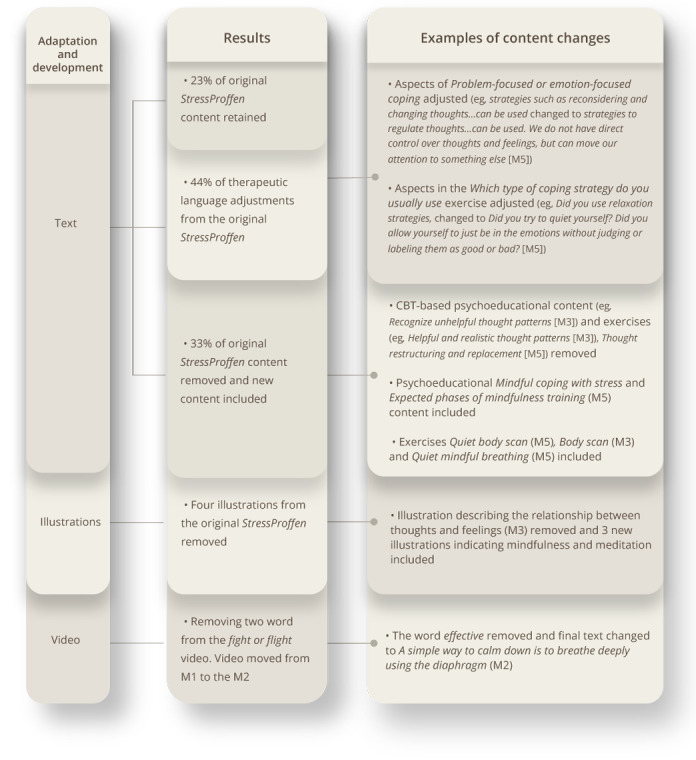
Overview—StressProffen MBI. Program content adaptation and development. CBT: cognitive behavioral therapy; M: module; MBI: mindfulness-based intervention.

General psychoeducational content was discussed and primarily kept in both versions (ie, CBI and MBI), with the MBI-version incorporating additional material for more mindfulness-based psychoeducational content. Of total content (ie, psychoeducational material, exercises, suggestions, and words of wisdom), 69% (76/110 steps) of the original *StressProffen* content were retained in the *StressProffen* CBI-version and 23% (28/123 steps) in the *StressProffen* MBI-version. Minor therapeutic language adjustments were conducted to adhere to the distinct style commonly used by CBT and mindfulness practitioners, respectively 25% (27/110 steps) for the CBI-version and 44% (54/123 steps) for the MBI-version. In addition, 6% (7/110 steps) of the original content was removed for the new CBI-version and 33% (41/123 steps) removed for the MBI-version. Adhering to the structure of the original *StressProffen* intervention, development of new mindfulness-based content was led by AM in collaboration with the core content development group. In this context, some content and exercises from Carlson and Speca’s MBIs [33] for coping with cancer was translated into Norwegian, adapted to the app format, and incorporated with permission from Carlson. Please see Figures 1 and 2 for an overview of program content adaptation and development.

To enable future program comparisons, both program versions were developed using the exact same *StressProffen* set-up and layout. The 2 final interventions therefore had the same basic structure and contained 10 modules with 9 to 16 steps each. While the MBI-version contained slightly more words (ie, total 33,020 vs total 30,134 for CBI) and lasted longer (approximately 6 hours compared to approximately 5 hours for CBI), the CBI-version contained more exercises (38 vs 30). These slight variances were deemed necessary for proper content presentation of each version and adhering to the basic tenants of CBT and mindfulness. MBIs typically revolve around fewer exercises than CBT, as excess variation could inhibit the development of mindfulness [31]. The reason for including some more text in the MBI was related to the need to explain some of the mindfulness exercises. For example, user tests (see next section) indicated that mindfulness exercises involving sitting in silence needed more explanations than CBI exercises.

The team otherwise strived to balance the number and length of modules and subsections, exercises, and figures as much as possible, and both versions allowed for setting reminders, marking favorite exercises and educational content, and for choosing between reading or listening. Please see Table 1 for a detailed overview of modules and steps in the 2 program versions.

**Table 1:** Overview of modules and steps in the CBI^a^ and MBI^b^ programs.

Module	CBI						MBI					
	Steps, n	Exercises, n	Words, n			Auditory (hh:mm:ss)	Steps, n	Exercises, n	Words, n			Auditory (hh:mm:ss)
			Total	Educational text	Exercises				Total	Educational text	Exercises	
1	11	2	3145	2049	1096	00:21:28	15	3	3993	2459	1534	00:32:47
2	10	3	2457	1356	1093	00:25:10	13	4	3966	2304	1662	00:52:23
3	11	3	2624	1861	754	00:25:34	16	3	4478	2754	1724	00:52:17
4	10	5	3199	1208	1985	00:34:48	9	2	2657	2110	547	00:22:49
5	12	5	2513	1217	1292	00:31:04	13^c^	4	3080	1713	1367	01:04:15^c^
6	10	3	2934	1292	1638	00:28:42	13	3	3575	2327	1244	00:31:51
7	9	4	3353	963	2384	00:37:09	9	3	3062	1280	1782	00:35:23
8	11	5	3340	1548	1788	00:32:31	10	3	2611	1746	861	00:21:07
9	12	4	3032	2132	898	00:32:08	12	2	3125	2433	690	00:29:31
10	14	4	3537	1713	1820	00:35:12	13	3	2473	1658	811	00:23:14
Total	110	38	30,134	15,339	14,748	05:03:46	123	30	33,020	20,784	12,222	06:05:37

^a^CBI: cognitive behavioral therapy-based intervention.

^b^MBI: mindfulness-based intervention.

^c^In 1 exercise, the participants could choose between spending 10 or 20 minutes on the exercise. This summary is based on a 10 minute exercise.

### User Testing of New Exercises

The newly developed exercises were audio-recorded prior to testing. User representatives (ie, 4 women with breast cancer) and 3 members of a project-related editorial team tested the new exercises in iterations. The user representatives stated that the opportunity to listen to the exercises, not only having to read, would be considered important, and that the type of voice (eg, calm and engaging) would be crucial for maintaining their interest for further program use. As the new mindfulness-based exercises also had silence (ie, quiet time) incorporated into the exercises, some of the users expressed experiencing some frustration with the silence, describing “loosing the flow” in the exercise when not hearing anything. Even a 5 second break was experienced as a long time for the users, and they stated that the silence could make thoughts occur related to their illness or situation. Silence is regarded an essential ingredient to receive the long term benefits of mindfulness, and could not be completely removed from the MBI version. However, this feedback from participants resulted in a more extended explanation before the exercises involving silence, and a shortening of some of the silent sections in the new mindfulness exercises. The users did describe silence in exercises focusing on bodily sensations (eg, “Silent body scan” and “Body scan”) as easier to accept, and the silence parts of these exercises were kept. The participating end users also stated that some of the new mindfulness-based exercises appeared to have abrupt endings. This feedback resulted in both versions (ie, CBI and MBI) incorporating the original *StressProffen* “closing sentence” (eg, “The exercise is now completed, and you are ready to move on to the next part”) at the end of all exercises.

### Technical and Static Aspects and Adjustments

The original *StressProffen* has an option for reading a “quote of the day” (ie, 55 different quotes) through using a specific button when opening the app. In the CBI-version, all quotes of the day were kept and 1 additional quote (ie, “We can easily manage if we will only take, each day, the burden appointed to it. But the load will be too heavy for us if we carry yesterday’s burden over again today, and then add the burden of the morrow before we are required to bear it”—John Newton) included. This quote was also added to the MBI-version. In the MBI-version, 11 original *StressProffen* quotes were removed, for example, to limit indications of self-judgement (eg, “Don’t underestimate yourself by comparing yourself to others. It’s our differences that make us unique” [Unknown]).

The original *StressProffen* also has 33 quotes or words of wisdom used as “additional reminders.” Of these, 8 were removed from the CBI-version due to mindfulness-related content, (eg, “To be mindful is to be grateful,” “To be mindful is to focus on the here and now”), and 5 were added (eg, “Don’t let the past or the future guide you, concentrate on the here and now”; “Allow yourself to smile or laugh, even when things are difficult”; “Think about it, what are you grateful for?”). In the MBI-version, 13 of the “additional reminders” were removed (eg, “Think of something that you can do before the day is over that gives meaning,” “To live your life the way you want to can make you stronger in body, mind and soul”) and 5 added (eg, “Can you observe your thoughts without evaluating them?”; “Take time to relax, or do something you like”; “Do things you like doing”).

**Figure 3:**
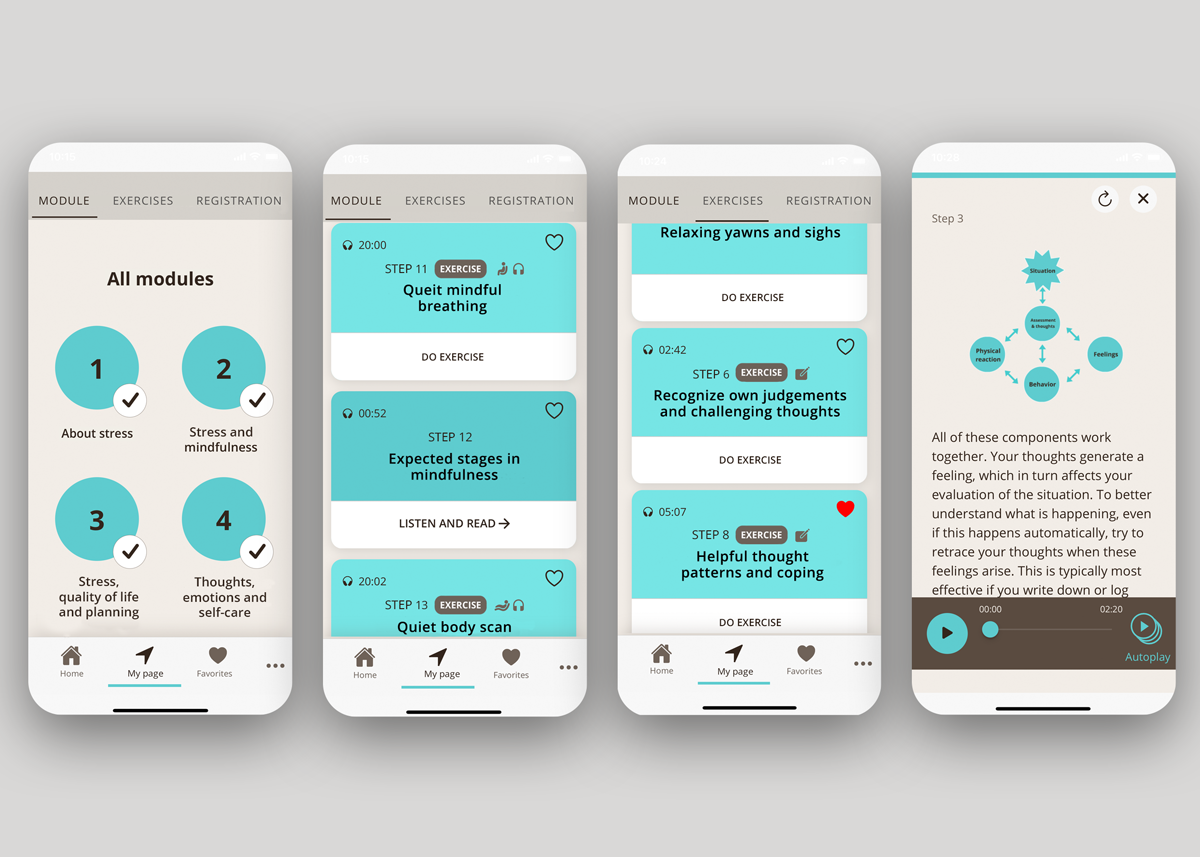
Examples of screenshots—StressProffen CBI- and MBI-versions. CBI: cognitive behavioral therapy-based intervention; MBI: mindfulness-based intervention.

A functionality to allow participants to choose the length of a mindfulness exercise (ie, 10 or 20 minutes) was also included in the MBI-version. Finally, to ensure the new app versions met with all ethical, privacy, and security requirements (eg, General Data Protection Regulation and Apple or Google requirements), a legal disclaimer and privacy protection details was developed and included aspects such as description of data collection, storage, user rights, and study contact information in case of questions or technical issues. See Figure 3 for screenshots from the CBI- and MBI-versions.

## Discussion

### Principal Findings

This study shows how theory specific digital stress-management interventions for survivors of breast cancer can be developed combining existing evidence and key stakeholder (eg, scientists, health care providers, and end user) input, centering around content adaptation and development, user testing and adjustment of technical, static, and privacy and security aspects. Findings show how the original *StressProffen* content was examined, overlapping content (ie, non-CBT or mindfulness specific) identified and retained. Mindfulness-based content was removed from the CBI-version, and cognitive behavioral content removed from the MBI-version, with varying degrees of new or adjusted content created for both versions, resulting in 2 distinct CBI and MBI interventions.

### Providing Options for Personal Stress-Management Preferences

CBT as well as MBIs may provide effective psychosocial support for survivors of cancer [11,12,27,29]. Studies comparing these types of digital self-management interventions are at best scarce, and this study provides a basis for conducting such comparisons. Presentation of content, controlling for format, display, content, and length, may as such allow for a true comparison of theoretical approaches (ie, CBT vs mindfulness).

Whether being able to choose between types of self-management interventions can in fact impact outcome remains to be seen, but people’s preference for treatment type, for example, type of psychotherapy vary [34,35], and user input does suggest that personal choice can increase motivation to engage in self-management, strengthening adherence, and potentially also impacting outcome. Studies matching patients’ treatment preferences with the intervention received, do not consistently impact treatment outcome however [36]. For example, a study examining the outcome of CBT versus MBIs in an RCT with participants diagnosed with social anxiety disorder did not find being assigned to personally preferred treatment, or not, to significantly impact treatment outcome [37]. Other studies have found the opposite to be the case. For example, 1 study comparing treatment program preferences in distressed survivors of breast cancer found a mindfulness-based cancer recovery intervention to be preferred over supportive expressive therapy or a 1-day stress-management seminar, and in an RCT found matching preference to treatment option, regardless of intervention received, to be effective when related to QoL and spiritual well-being [38]. Distinguishing between *having a choice* and actually *making a choice* may also be of interest, as 1 study found *having a choice* to be associated with more positive patient outcomes in terms of mood, patient satisfaction, perceived control, and less desire to seek further information than if having only 1 choice [39]. In the same study, actually *making a choice* appeared to have no impact on patient outcomes [39].

While CBT is often considered the “gold standard” for psychosocial stress-management interventions in cancer [27], a growing body of evidence supports the use of mindfulness for patients with cancer as well [11,12]. Which types of interventions work the best for which types of people, demographic variables, or cancer groups, however, remains to be seen. For people already having knowledge of, or known preference for, 1 theoretical approach (eg, preference for mindfulness or preference for CBT), actually being able to engage in this type of intervention could be of benefit for motivation, engagement, and intervention adherence, and subsequently also for future impact and outcome.

### Potential Impact for Future Research

The adaptation and development processes in this study strived to design the interventions in a way that the only differing factor would be whether a patient would be exposed to the active ingredients of CBT or mindfulness. Findings show how this goal was primarily achieved, despite the final MBI-version being slightly longer, with more steps and total words and auditory length compared to the final CBI-version, and the CBI-version having more exercises compared to the MBI-version. These variations are however minor, and could be justified in the differences of methodology (eg, necessity for more explanatory, guided exercises in CBT, and necessity for more elaborate wording related to “being present and nonjudgmental”—and the need for incorporating sections of silence in exercises—in mindfulness). Consequently, any future differential effects across the 2 interventions during comparison studies would likely be due to the mechanisms driving the effects of cognitive behavioral- and mindfulness-based methodology in survivors of cancer, rather than confounding factors such as design, layout, textual, or auditory length or other nontheoretical factors. Future research might also aim to incorporate other clinical therapeutic approaches in cancer, such as, for example, acceptance and commitment therapy [40], for exploration in digital interventions. Analysis of usage patterns could also add to knowledge about how the different programs are implemented and used over time. As various formats and visual presentations created across study teams could impact interest in, and actual use of, interventions, comparing result across studies and research teams might prove difficult, however. Future studies embarking on a similar challenge should nevertheless keep striving to make the interventions as similar as possible while retaining the differences in mechanisms.

The original *StressProffen* intervention is primarily CBT-based, but also contains options for reminders in the form of inspirational quotes, as well as the choice of sequence between modules 5 to 9, the possibility to register daily stress levels, and the option to mark favorite exercises in a “my page” section [22]. These options were retained in both new versions, offering additional choices in support of personal preferences. Stakeholder input in this study, including from survivors of breast cancer and oncology health care providers, supported previous research underlining the importance of being able to choose between reading or listening [22,23]. Findings also pointed to a need for clear explanations for sections that might not be self-explanatory, such as, for example, areas of silence in the mindfulness-based version, underlining the need for interventions to be tailored to the patients’ needs [41].

Posttesting the asynchronous CBI and MBI versions in a clinical trial, future research could also consider building therapist-guided aspects into the interventions. As such, including synchronous components to asynchronous platforms could potentially enhance the effect and make the intervention more known to patients. Given the importance of health economic aspects in health care and health care research, health economy should also be evaluated to inform future implementation into regular practice.

### Limitations

This study has a number of limitations. First, user testing was performed by only 4 survivors of breast cancer. However, the original *StressProffen* intervention was developed with user input, enabling user testing of primarily new exercises in the 2 new versions. In addition, project team members, including health care providers, also conducted extensive testing of the new CBI and MBI versions.

Second, although striving to keep the 2 interventions equal in structure and length, the MBI version ended up including somewhat more extensive content, with more steps and words, and the audio files subsequently lasting longer than those in the CBI version. Some of this can be explained by the nature of MBI, with more silence in the audio-files needing explanations, but a potential impact of this difference in length should be examined in future studies comparing the 2 distinct intervention versions.

Third, some of the adaption and development of program content were conducted during the COVID-19 pandemic, meaning that several workshops had to be conducted digitally due to pandemic restrictions. This could potentially have impacted the development process. However, the core and expanded content development groups had met in person several times prior to the pandemic, making the productivity and ease of digital meetings acceptable. Collaborating on text evaluation and progress was also easily conducted first through email and subsequently through digital discussions.

### Future Directions

This study sought to describe a method for developing distinct digital psychosocial stress-management interventions based on an existing evidence-based, user-centered intervention. Future studies should examine whether distinct interventions based on CBT and mindfulness can have positive impact for survivors of breast cancer, and an RCT exploring such impact (ie, through the research project *Coping After Breast Cancer*, Principal Investigator Giske Ursin of the Norwegian Cancer Registry) is currently underway [42]. In the future, if found to be of equivalent usefulness, potential, and impact, patients with cancer could opt to choose between CBT and mindfulness-based digital psychosocial stress-management programs, based on preferences. Potential impact of receiving the preferred intervention or not should however also be explored.

### Conclusions

This study shows how theory specific digital stress-management interventions for survivors of breast cancer can be developed through extensive collaborations between key stakeholders with the right expertise. In this case, including scientists, health care providers, and survivors of breast cancer. Offering a variety of digital evidence-informed stress-management approaches may increase interest for, as well as outreach of, and potentially also impact of, psychosocial interventions for survivors of cancer.

## Abbreviations

CBI: cognitive behavioral therapy–based intervention
CBT: cognitive behavioral therapy
MBI: mindfulness-based intervention
QoL: quality of life
RCT: randomized control trial

## References

[1] Stanton AL (2006). Psychosocial concerns and interventions for cancer survivors. J Clin Oncol.

[2] Stein KD, Syrjala KL, Andrykowski MA (2008). Physical and psychological long-term and late effects of cancer. Cancer.

[3] Mitchell AJ, Chan M, Bhatti H, Halton M, Grassi L, Johansen C, Meader N (2011). Prevalence of depression, anxiety, and adjustment disorder in oncological, haematological, and palliative-care settings: a meta-analysis of 94 interview-based studies. Lancet Oncol.

[4] Stanton AL (2012). What happens now? Psychosocial care for cancer survivors after medical treatment completion. J Clin Oncol.

[5] Ehlers SL, Davis K, Bluethmann SM, Quintiliani LM, Kendall J, Ratwani RM, Diefenbach MA, Graves KD (2019). Screening for psychosocial distress among patients with cancer: implications for clinical practice, healthcare policy, and dissemination to enhance cancer survivorship. Transl Behav Med.

[6] Andersen BL (1992). Psychological interventions for cancer patients to enhance the quality of life. J Consult Clin Psychol.

[7] Antoni MH, Lehman JM, Kilbourn KM, Boyers AE, Culver JL, Alferi SM, Yount SE, McGregor BA, Arena PL, Harris SD, Price AA, Carver CS (2001). Cognitive-behavioral stress management intervention decreases the prevalence of depression and enhances benefit finding among women under treatment for early-stage breast cancer. Health Psychol.

[8] Manne SL, Andrykowski MA (2006). Are psychological interventions effective and accepted by cancer patients? II. Using empirically supported therapy guidelines to decide. Ann Behav Med.

[9] Gudenkauf LM, Ehlers SL (2018). Psychosocial interventions in breast cancer survivorship care. Breast.

[10] Shennan C, Payne S, Fenlon D (2011). What is the evidence for the use of mindfulness-based interventions in cancer care? A review. Psychooncology.

[11] Haller H, Winkler MM, Klose P, Dobos G, Kümmel S, Cramer H (2017). Mindfulness-based interventions for women with breast cancer: an updated systematic review and meta-analysis. Acta Oncol.

[12] Cillessen L, Johannsen M, Speckens AEM, Zachariae R (2019). Mindfulness-based interventions for psychological and physical health outcomes in cancer patients and survivors: a systematic review and meta-analysis of randomized controlled trials. Psychooncology.

[13] Carlson LE (2016). Mindfulness-based interventions for coping with cancer. Ann N Y Acad Sci.

[14] Rummans TA, Clark MM, Sloan JA, Frost MH, Bostwick JM, Atherton PJ, Johnson ME, Gamble G, Richardson J, Brown P, Martensen J, Miller J, Piderman K, Huschka M, Girardi J, Hanson J (2006). Impacting quality of life for patients with advanced cancer with a structured multidisciplinary intervention: a randomized controlled trial. J Clin Oncol.

[15] Buneviciene I, Mekary RA, Smith TR, Onnela J, Bunevicius A (2021). Can mHealth interventions improve quality of life of cancer patients? A systematic review and meta-analysis. Crit Rev Oncol Hematol.

[16] McAlpine H, Joubert L, Martin-Sanchez F, Merolli M, Drummond KJ (2015). A systematic review of types and efficacy of online interventions for cancer patients. Patient Educ Couns.

[17] Seiler A, Klaas V, Tröster G, Fagundes CP (2017). eHealth and mHealth interventions in the treatment of fatigued cancer survivors: a systematic review and meta-analysis. Psychooncology.

[18] Chambers SK, Ritterband LM, Thorndike F, Nielsen L, Aitken JF, Clutton S, Scuffham PA, Youl P, Morris B, Baade PD, Dunn J (2018). Web-delivered cognitive behavioral therapy for distressed cancer patients: randomized controlled trial. J Med Internet Res.

[19] Penedo FJ, Oswald LB, Kronenfeld JP, Garcia SF, Cella D, Yanez B (2020). The increasing value of eHealth in the delivery of patient-centred cancer care. Lancet Oncol.

[20] Denecke K, Schmid N, Nüssli S (2022). Implementation of cognitive behavioral therapy in e-Mental health apps: literature review. J Med Internet Res.

[21] Matis J, Svetlak M, Slezackova A, Svoboda M, Šumec R (2020). Mindfulness-based programs for patients with cancer via eHealth and mobile health: systematic review and synthesis of quantitative research. J Med Internet Res.

[22] Børøsund E, Mirkovic J, Clark MM, Ehlers SL, Andrykowski MA, Bergland A, Westeng M, Nes LS (2018). A stress management app intervention for cancer survivors: design, development, and usability testing. JMIR Form Res.

[23] Børøsund E, Varsi C, Clark MM, Ehlers SL, Andrykowski MA, Sleveland HRS, Bergland A, Nes LS (2020). Pilot testing an app-based stress management intervention for cancer survivors. Transl Behav Med.

[24] Børøsund E, Ehlers SL, Varsi C, Clark MM, Andrykowski MA, Cvancarova M, Nes LS (2020). Results from a randomized controlled trial testing StressProffen; an application-based stress-management intervention for cancer survivors. Cancer Med.

[25] Børøsund E, Ehlers SL, Clark MM, Andrykowski MA, Småstuen MC, Nes LS (2022). Digital stress management in cancer: testing StressProffen in a 12-month randomized controlled trial. Cancer.

[26] Antoni M, Smith R (2003). Stress Management Intervention for Women with Breast Cancer.

[27] Hulbert-Williams NJ, Beatty L, Dhillon HM (2018). Psychological support for patients with cancer: evidence review and suggestions for future directions. Curr Opin Support Palliat Care.

[28] Zeichner SB, Zeichner RL, Gogineni K, Shatil S, Ioachimescu O (2017). Cognitive behavioral therapy for insomnia, mindfulness, and yoga in patients with breast cancer with sleep disturbance: a literature review. Breast Cancer (Auckl).

[29] Möller UO, Beck I, Rydén L, Malmström M (2019). A comprehensive approach to rehabilitation interventions following breast cancer treatment—a systematic review of systematic reviews. BMC Cancer.

[30] Garland SN, Mahon K, Irwin MR (2019). Integrative approaches for sleep health in cancer survivors. Cancer J.

[31] Grossman P, Niemann L, Schmidt S, Walach H (2004). Mindfulness-based stress reduction and health benefits. A meta-analysis. J Psychosom Res.

[32] Zhang L, Lopes S, Lavelle T, Jones KO, Chen L, Jindal M, Zinzow H, Shi L (2022). Economic evaluations of mindfulness-based interventions: a systematic review. Mindfulness (N Y).

[33] Carlson LE, Speca M (2011). Mindfulness-Based Cancer Recovery: A Step-by-Step MBSR Approach to Help You Cope with Treatment and Reclaim Your Life.

[34] Sandell R, Clinton D, Frövenholt J, Bragesjö M (2011). Credibility clusters, preferences, and helpfulness beliefs for specific forms of psychotherapy. Psychol Psychother.

[35] Williams R, Farquharson L, Palmer L, Bassett P, Clarke J, Clark DM, Crawford MJ (2016). Patient preference in psychological treatment and associations with self-reported outcome: national cross-sectional survey in England and Wales. BMC Psychiatry.

[36] Windle E, Tee H, Sabitova A, Jovanovic N, Priebe S, Carr C (2020). Association of patient treatment preference with dropout and clinical outcomes in adult psychosocial mental health interventions: a systematic review and meta-analysis. JAMA Psychiatry.

[37] Koszycki D, Ilton J, Dowell A, Bradwejn J (2022). Does treatment preference affect outcome in a randomized trial of a mindfulness intervention versus cognitive behaviour therapy for social anxiety disorder?. Clin Psychol Psychother.

[38] Carlson LE, Tamagawa R, Stephen J, Doll R, Faris P, Dirkse D, Speca M (2014). Tailoring mind-body therapies to individual needs: patients' program preference and psychological traits as moderators of the effects of mindfulness-based cancer recovery and supportive-expressive therapy in distressed breast cancer survivors. J Natl Cancer Inst Monogr.

[39] Ogden J, Daniells E, Barnett J (2009). When is choice a good thing? An experimental study of the impact of choice on patient outcomes. Psychol Health Med.

[40] Hulbert-Williams NJ, Storey L, Wilson KG (2015). Psychological interventions for patients with cancer: psychological flexibility and the potential utility of acceptance and commitment therapy. Eur J Cancer Care (Engl).

[41] Dineen-Griffin S, Garcia-Cardenas V, Williams K, Benrimoj SI (2019). Helping patients help themselves: a systematic review of self-management support strategies in primary health care practice. PLoS One.

[42] Svendsen K, Nes LS, Meland A, Larsson IM, Gjelsvik YM, Børøsund E, Rygg CM, Myklebust TÅ, Reinertsen KV, Kiserud CE, Skjerven H, Antoni MH, Chalder T, Mjaaland I, Carlson LE, Eriksen HR, Ursin G (2023). Coping after breast cancer: protocol for a randomized controlled trial of stress management eHealth interventions. JMIR Res Protoc.

